# The genome sequence of the dark-edged bee fly, *Bombylius major *(Linnaeus, 1758)

**DOI:** 10.12688/wellcomeopenres.19804.1

**Published:** 2023-09-01

**Authors:** Mara K.N. Lawniczak, Liam M. Crowley, Erica McAlister, University of Oxford and Wytham Woods Genome Acquisition Lab, Darwin Tree of Life Barcoding collective, Wellcome Sanger Institute Tree of Life programme, Wellcome Sanger Institute Scientific Operations: DNA Pipelines collective, Tree of Life Core Informatics collective, Darwin Tree of Life Consortium

**Affiliations:** 1 Tree of Life, Wellcome Sanger Institute, Hinxton, England, UK; 2 Department of Biology, University of Oxford, Oxford, England, UK; 3 Natural History Museum, London, England, UK

**Keywords:** Bombylius major, dark-edged bee fly, genome sequence, chromosomal, Diptera

## Abstract

We present a genome assembly from an individual male *Bombylius major* (the dark-edged bee fly; Arthropoda; Insecta; Diptera; Bombyliidae). The genome sequence is 304.3 megabases in span. The whole assembly is scaffolded into 7 chromosomal pseudomolecules, including the X and Y sex chromosomes. The mitochondrial genome has also been assembled and is 17.8 kilobases in length. Gene annotation of this assembly on Ensembl identified 10,852 protein coding genes.

## Species taxonomy

Eukaryota; Metazoa; Eumetazoa; Bilateria; Protostomia; Ecdysozoa; Panarthropoda; Arthropoda; Mandibulata; Pancrustacea; Hexapoda; Insecta; Dicondylia; Pterygota; Neoptera; Endopterygota; Diptera; Brachycera; Muscomorpha; Asiloidea; Bombyliidae; Bombyliinae; *Bombylius*; *Bombylius major* (Linnaeus, 1758) (NCBI:txid240869).

## Background

**Figure 1.**
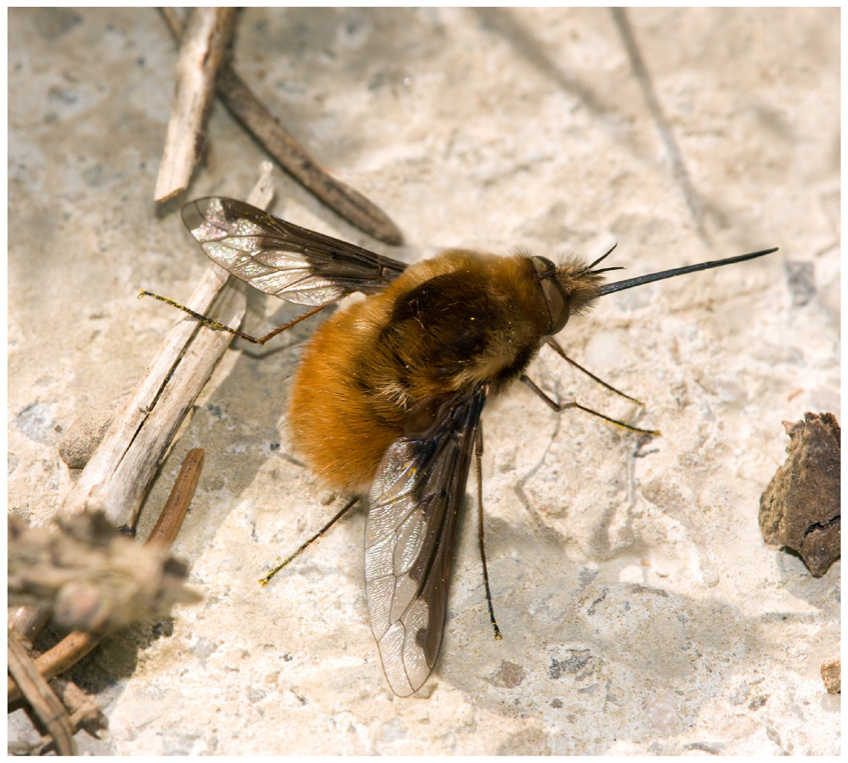
Photograph of *Bombylius major* gathering nectar from a flower (Photograph by Richard Bartz, Munich aka Makro Freak CC-BY-SA 2.5).

The dark-edged bee fly, *Bombylius major*, is an important pollinator with a large range across North America, Europe, and Asia. *Bombylius major* is a medium-large bee fly, with distinctive dark brown markings on the front edge of its wings when seen at rest. The long proboscis is characteristic of this genus. Males have holoptic eyes that meet at the top but both adult male and female are hairy, including their heads, although it has been noted that often the females have paler body hairs ( Stubbs & Drake, 2014) – a common name of fluffy flying narwhals is very apt ( Figure 1). Both sexes have slender legs terminating in equally slender, sharply curled claws ( Hull, 1973). Males are thought to exhibit some territorial behaviour when courting. The females of *B. major*, like many other bee fly species, have specialised “sand chambers” or “dust baskets” at the end of their abdomen where they gather sand to coat their eggs prior to flicking them into the burrow entrances of bees ( Stubbs & Drake, 2014).

Eggs are small (0.5 mm), translucent and numerous and are scattered in a ‘speculative’ manner ( Stubbs & Drake, 2014). The first instar larvae are planidial, differing from the other instars in being a highly active host-seeking stage. It is elongated with a few bristles on the body used to aid locomotion. By the third instar stage, the larvae have changed to a grub-like form having greatly increased in mass but no longer actively moving. The larvae are ectoparasitoids of solitary bees, *Andrena* spp. The final stages of the pupa are active, enabling the bee fly to leave the host’s nest and come to the surface before the adults emerge. These are some of the first adult insects that we see in spring in the UK and announce that the cold winter days are over!

## Genome sequence report

The genome was sequenced from one male *Bombylius major* collected from Saffron Walden, UK (52.02, 0.25). A total of 66-fold coverage in Pacific Biosciences single-molecule HiFi long reads and 127-fold coverage in 10X Genomics read clouds was generated. Primary assembly contigs were scaffolded with chromosome conformation Hi-C data. Manual assembly curation corrected 101 missing joins or mis-joins and removed one haplotypic duplication, reducing the scaffold number by 40.72%.

**Figure 2.**
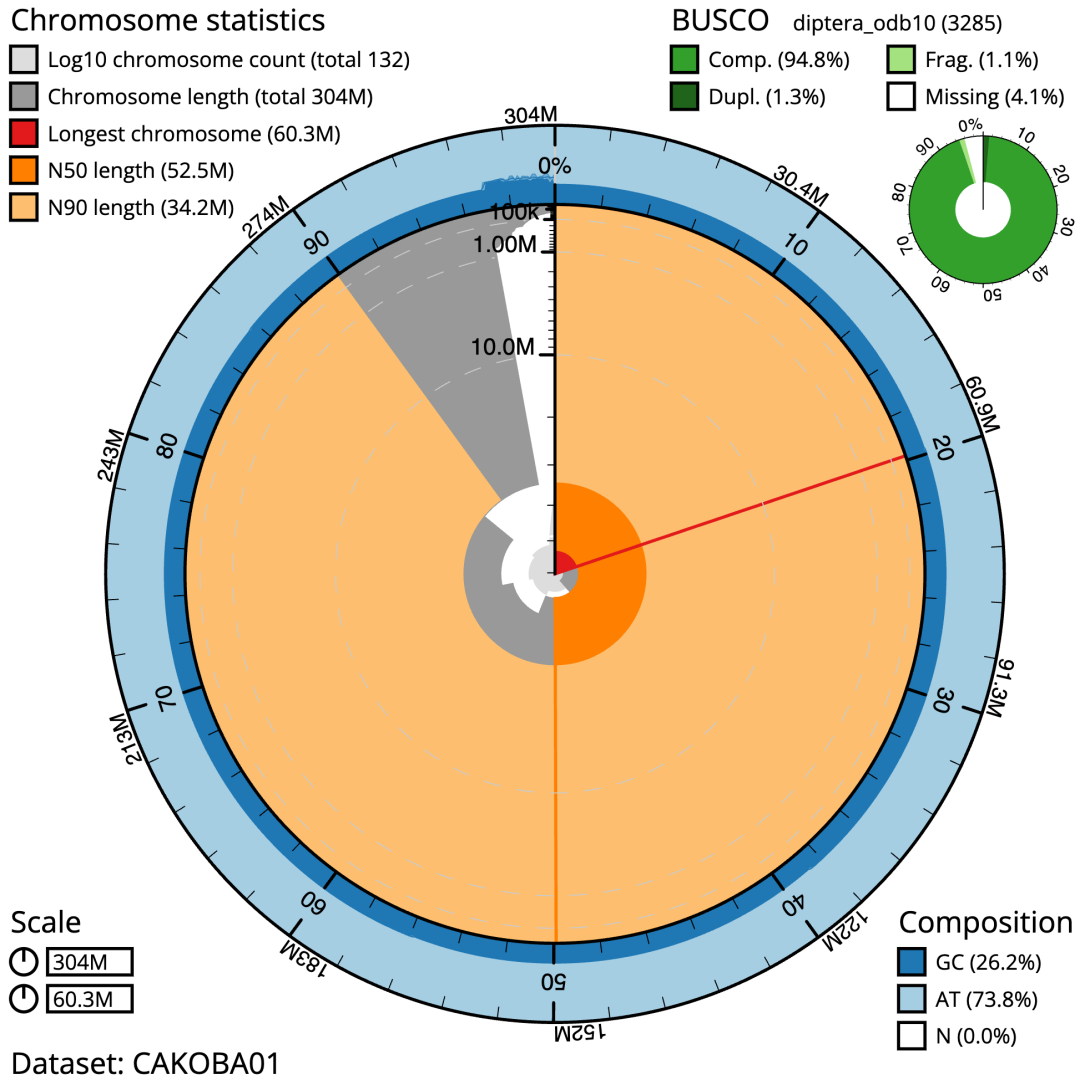
Genome assembly of *Bombylius major*, idBomMajo1.1: metrics. The BlobToolKit Snailplot shows N50 metrics and BUSCO gene completeness. The main plot is divided into 1,000 size-ordered bins around the circumference with each bin representing 0.1% of the 304,329,675 bp assembly. The distribution of scaffold lengths is shown in dark grey with the plot radius scaled to the longest scaffold present in the assembly (60,331,320 bp, shown in red). Orange and pale-orange arcs show the N50 and N90 scaffold lengths (52,534,525 and 34,171,463 bp), respectively. The pale grey spiral shows the cumulative scaffold count on a log scale with white scale lines showing successive orders of magnitude. The blue and pale-blue area around the outside of the plot shows the distribution of GC, AT and N percentages in the same bins as the inner plot. A summary of complete, fragmented, duplicated and missing BUSCO genes in the diptera_odb10 set is shown in the top right. An interactive version of this figure is available at https://blobtoolkit.genomehubs.org/view/idBomMajo1.1/dataset/CAKOBA01/snail.

The final assembly has a total length of 304.3 Mb in 131 sequence scaffolds with a scaffold N50 of 52.5 Mb ( Table 1). The whole assembly sequence was assigned to 7 chromosomal-level scaffolds, representing 5 autosomes and the X and Y sex chromosome. Chromosome-scale scaffolds confirmed by the Hi-C data are named in order of size ( Figure 2– Figure 5; Table 2). The order and orientation of scaffolds is uncertain on chromosome X in the region 13.5–18.1 Mb. While not fully phased, the assembly deposited is of one haplotype. Contigs corresponding to the second haplotype have also been deposited. The mitochondrial genome was also assembled and can be found as a contig within the multifasta file of the genome submission.

**Table 1.** Genome data for *Bombylius major*, idBomMajo1.1.

Project accession data		
Assembly identifier	idBomMajo1.1	
Species	*Bombylius major*	
Specimen	idBomMajo1	
NCBI taxonomy ID	240869	
BioProject	PRJEB50855	
BioSample ID	SAMEA7524251	
Isolate information	idBomMajo1, male: thorax (DNA sequencing and Hi-C scaffolding) idBomMajo2, abdomen (RNA sequencing)	
Assembly metrics *		*Benchmark*
Consensus quality (QV)	54.3	*≥ 50*
*k*-mer completeness	99.99%	*≥ 95%*
BUSCO **	C:94.8%[S:93.5%,D:1.3%], F:1.1%,M:4.1%,n:3,285	*C ≥ 95%*
Percentage of assembly mapped to chromosomes	100%	*≥ 95%*
Sex chromosomes	X and Y chromosomes	*localised homologous pairs*
Organelles	Mitochondrial genome assembled	*complete single alleles*
Raw data accessions		
PacificBiosciences SEQUEL II	ERR8705853	
10X Genomics Illumina	ERR8571707–ERR8571710	
Hi-C Illumina	ERR8571711	
PolyA RNA-Seq Illumina	ERR10123677	
Genome assembly		
Assembly accession	GCA_932526495.1	
*Accession of alternate haplotype*	GCA_932526615.1	
Span (Mb)	304.3	
Number of contigs	241	
Contig N50 length (Mb)	18.8	
Number of scaffolds	131	
Scaffold N50 length (Mb)	52.5	
Longest scaffold (Mb)	60.33	
Genome annotation		
Number of protein-coding genes	10,852	
Number of non-coding genes	1,444	
Number of gene transcripts	17,940	

* Assembly metric benchmarks are adapted from column VGP-2020 of “Table 1: Proposed standards and metrics for defining genome assembly quality” from ( Rhie *et al.*, 2021). ** BUSCO scores based on the diptera_odb10 BUSCO set using v5.3.2. C = complete [S = single copy, D = duplicated], F = fragmented, M = missing, n = number of orthologues in comparison. A full set of BUSCO scores is available at https://blobtoolkit.genomehubs.org/view/idBomMajo1.1/dataset/CAKOBA01/busco.

**Figure 3.**
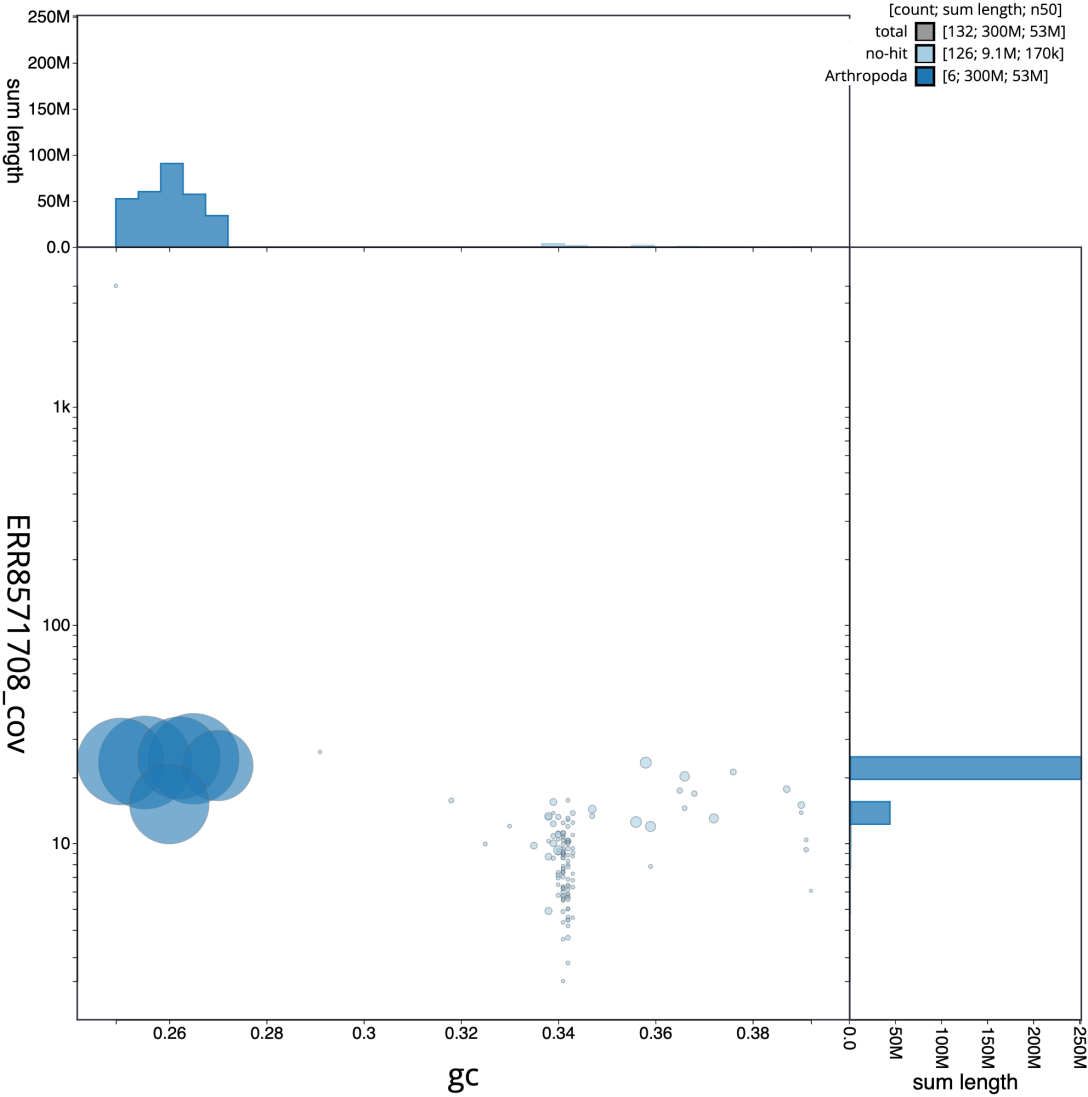
Genome assembly of *Bombylius major*, idBomMajo1.1: BlobToolKit GC-coverage plot. Scaffolds are coloured by phylum. Circles are sized in proportion to scaffold length. Histograms show the distribution of scaffold length sum along each axis. An interactive version of this figure is available at https://blobtoolkit.genomehubs.org/view/idBomMajo1.1/dataset/CAKOBA01/blob.

**Figure 4.**
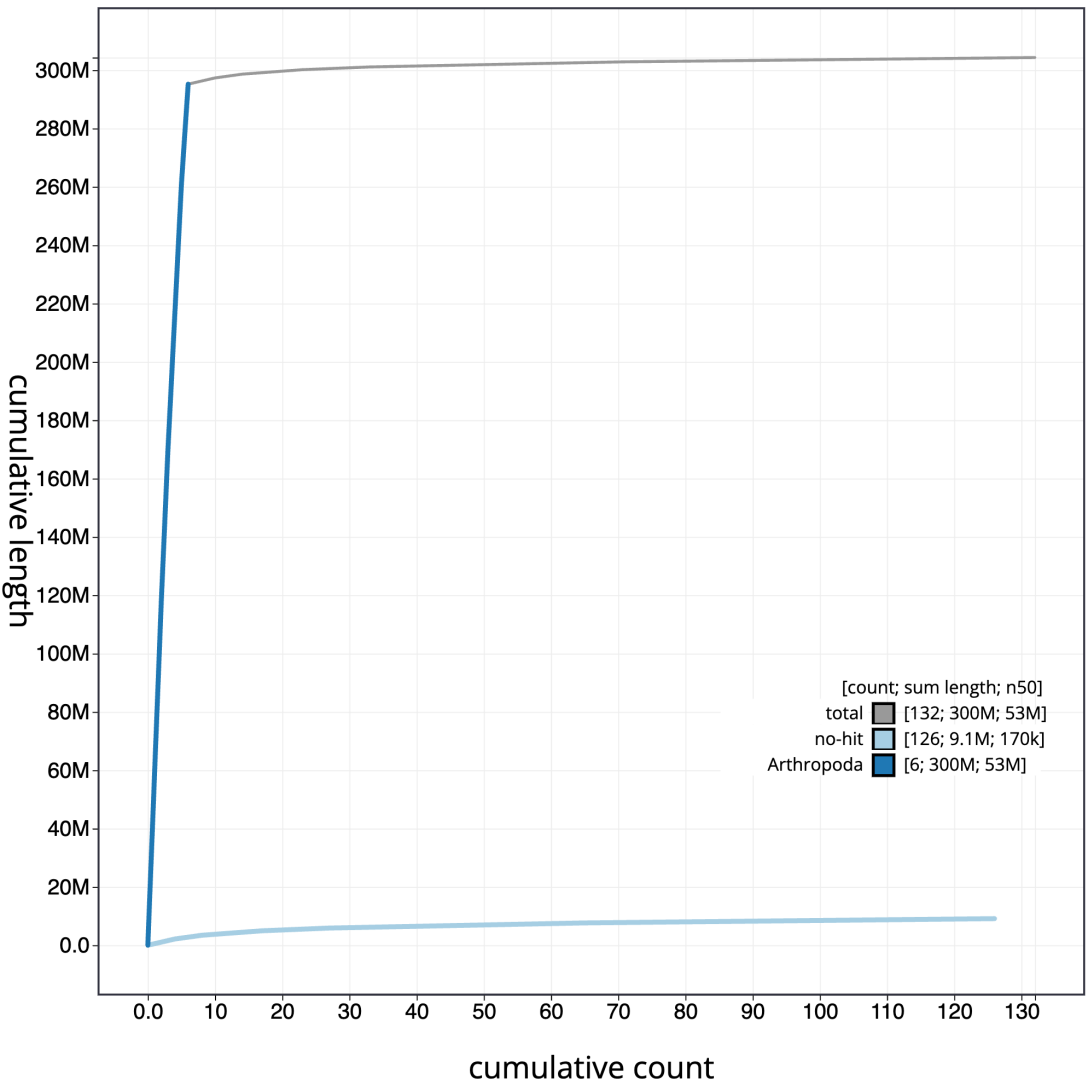
Genome assembly of *Bombylius major*, idBomMajo1.1: BlobToolKit cumulative sequence plot. The grey line shows cumulative length for all scaffolds. Coloured lines show cumulative lengths of scaffolds assigned to each phylum using the buscogenes taxrule. An interactive version of this figure is available at https://blobtoolkit.genomehubs.org/view/idBomMajo1.1/dataset/CAKOBA01/cumulative.

**Figure 5.**
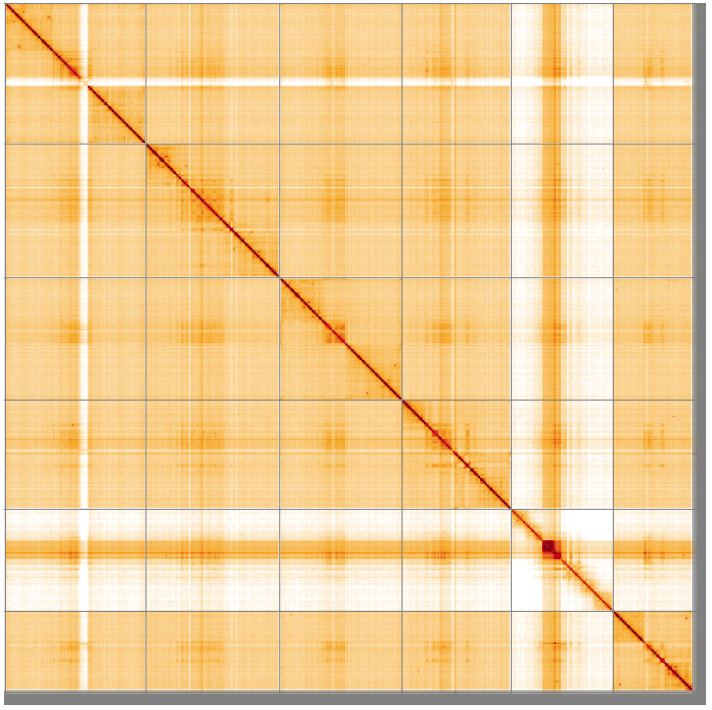
Genome assembly of *Bombylius major*, idBomMajo1.1: Hi-C contact map of the idBomMajo1.1 assembly, visualised using HiGlass. Chromosomes are shown in order of size from left to right and top to bottom. An interactive version of this figure may be viewed at https://genome-note-higlass.tol.sanger.ac.uk/l/?d=TZRiIY3jQkqzJFFQmTndzQ.

**Table 2.** Chromosomal pseudomolecules in the genome assembly of *Bombylius major*, idBomMajo1.

INSDC accession	Chromosome	Length (Mb)	GC%
OW052042.1	1	60.33	25.5
OW052043.1	2	57.43	26.5
OW052044.1	3	52.53	25.0
OW052045.1	4	46.93	26.0
OW052047.1	5	34.17	27.0
OW052046.1	X	43.81	26.0
OW052048.1	Y	0.62	36.0
OW052049.1	MT	0.02	25.0

The estimated Quality Value (QV) of the final assembly is 54.3 with *k*-mer completeness of 99.99%, and the assembly has a BUSCO v5.3.2 completeness of 94.8% (single = 93.5%, duplicated = 1.3%), using the diptera_odb10 reference set ( *n* = 3,285).

Metadata for specimens, spectral estimates, sequencing runs, contaminants and pre-curation assembly statistics can be found at https://links.tol.sanger.ac.uk/species/240869.

## Genome annotation report

The *Bombylius major* genome assembly (GCA_932526495.1) was annotated using the Ensembl rapid annotation pipeline ( Table 1; https://rapid.ensembl.org/Bombylius_major_GCA_932526495.1/Info/Index). The resulting annotation includes 17,940 transcribed mRNAs from 10,852 protein-coding and 1,444 non-coding genes.

## Methods

### Sample acquisition and nucleic acid extraction

The specimen used for genome sequencing and Hi-C scaffolding was a male *Bombylius major* (specimen ID SAN0001229, individual idBomMajo1), which was netted in a garden in Saffron Walden, UK (latitude 52.02, longitude 0.25) on 2020-04-06. The specimen was collected and identified by Mara Lawniczak (Wellcome Sanger Institute), and was snap-frozen on dry ice.

The specimen used for RNA sequencing (specimen ID Ox001111, individual idBomMajo2) was collected from Wytham Woods, Oxfordshire (biological vice-county Berkshire), UK (latitude 51.77, longitude –1.31) by Liam Crowley (University of Oxford).

DNA was extracted at the Tree of Life laboratory, Wellcome Sanger Institute (WSI). The idBomMajo1 sample was weighed and dissected on dry ice with tissue set aside for Hi-C sequencing. Thorax tissue was disrupted using a Nippi Powermasher fitted with a BioMasher pestle *.* High molecular weight (HMW) DNA was extracted using the Qiagen MagAttract HMW DNA extraction kit. Low molecular weight DNA was removed from a 20 ng aliquot of extracted DNA using the 0.8X AMpure XP purification kit prior to 10X Chromium sequencing; a minimum of 50 ng DNA was submitted for 10X sequencing. HMW DNA was sheared into an average fragment size of 12–20 kb in a Megaruptor 3 system with speed setting 30. Sheared DNA was purified by solid-phase reversible immobilisation using AMPure PB beads with a 1.8X ratio of beads to sample to remove the shorter fragments and concentrate the DNA sample. The concentration of the sheared and purified DNA was assessed using a Nanodrop spectrophotometer and Qubit Fluorometer and Qubit dsDNA High Sensitivity Assay kit. Fragment size distribution was evaluated by running the sample on the FemtoPulse system.

RNA was extracted from the abdomen tissue of idBomMajo2 in the Tree of Life Laboratory at the WSI using TRIzol, according to the manufacturer’s instructions. RNA was then eluted in 50 μl RNAse-free water and its concentration assessed using a Nanodrop spectrophotometer and Qubit Fluorometer using the Qubit RNA Broad-Range (BR) Assay kit. Analysis of the integrity of the RNA was done using Agilent RNA 6000 Pico Kit and Eukaryotic Total RNA assay.

### Sequencing

Pacific Biosciences HiFi circular consensus and 10X Genomics read cloud DNA sequencing libraries were constructed according to the manufacturers’ instructions. Poly(A) RNA-Seq libraries were constructed using the NEB Ultra II RNA Library Prep kit. DNA and RNA sequencing were performed by the Scientific Operations core at the WSI on Pacific Biosciences SEQUEL II (HiFi), Illumina NovaSeq 6000 (RNA-Seq) and HiSeq X Ten (10X) instruments. Hi-C data were also generated from thorax tissue of idBomMajo1 that had been set aside, using the Arima2 kit and sequenced on the Illumina NovaSeq 6000 instrument.

### Genome assembly, curation and evaluation

Assembly was carried out with Hifiasm ( Cheng *et al.*, 2021) and haplotypic duplication was identified and removed with purge_dups ( Guan *et al.*, 2020). One round of polishing was performed by aligning 10X Genomics read data to the assembly with Long Ranger ALIGN, calling variants with FreeBayes ( Garrison & Marth, 2012). The assembly was then scaffolded with Hi-C data ( Rao *et al.*, 2014) using YaHS. The assembly was checked for contamination and corrected as described previously ( Howe *et al.*, 2021). Manual curation was performed using HiGlass ( Kerpedjiev *et al.*, 2018) and Pretext ( Harry, 2022). The mitochondrial genome was assembled using MitoHiFi ( Uliano-Silva *et al.*, 2023), which runs MitoFinder ( Allio *et al.*, 2020) or MITOS ( Bernt *et al.*, 2013) and uses these annotations to select the final mitochondrial contig and to ensure the general quality of the sequence.

A Hi-C map for the final assembly was produced using bwa-mem2 ( Vasimuddin *et al.*, 2019) in the Cooler file format ( Abdennur & Mirny, 2020). To assess the assembly metrics, the *k*-mer completeness and QV consensus quality values were calculated in Merqury ( Rhie *et al.*, 2020). This work was done using Nextflow ( Di Tommaso *et al.*, 2017) DSL2 pipelines “sanger-tol/readmapping” ( Surana *et al.*, 2023a) and “sanger-tol/genomenote” ( Surana *et al.*, 2023b). The genome was analysed within the BlobToolKit environment ( Challis *et al.*, 2020) and BUSCO scores ( Manni *et al.*, 2021; Simão *et al.*, 2015) were calculated.

Table 3 contains a list of relevant software tool versions and sources.

**Table 3.** Software tools: versions and sources.

Software tool	Version	Source
BlobToolKit	4.1.5	https://github.com/blobtoolkit/blobtoolkit
BUSCO	5.3.2	https://gitlab.com/ezlab/busco
FreeBayes	1.3.1-17-gaa2ace8	https://github.com/freebayes/freebayes
Hifiasm	0.15.3	https://github.com/chhylp123/hifiasm
HiGlass	1.11.6	https://github.com/higlass/higlass
Long Ranger ALIGN	2.2.2	https://support.10xgenomics.com/genome-exome/ software/pipelines/latest/advanced/other-pipelines
Merqury	MerquryFK	https://github.com/thegenemyers/MERQURY.FK
MitoHiFi	2	https://github.com/marcelauliano/MitoHiFi
PretextView	0.2	https://github.com/wtsi-hpag/PretextView
purge_dups	1.2.3	https://github.com/dfguan/purge_dups
sanger-tol/genomenote	v1.0	https://github.com/sanger-tol/genomenote
sanger-tol/readmapping	1.1.0	https://github.com/sanger-tol/readmapping/tree/1.1.0
YaHS	1.0	https://github.com/c-zhou/yahs

### Genome annotation

The Ensembl gene annotation system ( Aken *et al.*, 2016) was used to generate annotation for the *Bombylius major* assembly (GCA_932526495.1). Annotation was created primarily through alignment of transcriptomic data to the genome, with gap filling via protein-to-genome alignments of a select set of proteins from UniProt ( UniProt Consortium, 2019).

### Wellcome Sanger Institute – Legal and Governance

The materials that have contributed to this genome note have been supplied by a Tree of Life collaborator. The Wellcome Sanger Institute employs a process whereby due diligence is carried out proportionate to the nature of the materials themselves, and the circumstances under which they have been/are to be collected and provided for use. The purpose of this is to address and mitigate any potential legal and/or ethical implications of receipt and use of the materials as part of the research project, and to ensure that in doing so we align with best practice wherever possible. The overarching areas of consideration are:

- Ethical review of provenance and sourcing of the material
- Legality of collection, transfer and use (national and international)

Each transfer of samples is undertaken according to a Research Collaboration Agreement or Material Transfer Agreement entered into by the Tree of Life collaborator, Genome Research Limited (operating as the Wellcome Sanger Institute) and in some circumstances other Tree of Life collaborators.

## Data Availability

European Nucleotide Archive: *Bombylius major* (dark-edged bee fly). Accession number PRJEB50855; https://identifiers.org/ena.embl/PRJEB50855. ( Wellcome Sanger Institute, 2022) The genome sequence is released openly for reuse. The *Bombylius major* genome sequencing initiative is part of the Darwin Tree of Life (DToL) project. All raw sequence data and the assembly have been deposited in INSDC databases. Raw data and assembly accession identifiers are reported in Table 1.

## Author information

Members of the University of Oxford and Wytham Woods Genome Acquisition Lab are listed here: https://doi.org/10.5281/zenodo.4789928.

Members of the Darwin Tree of Life Barcoding collective are listed here: https://doi.org/10.5281/zenodo.4893703.

Members of the Wellcome Sanger Institute Tree of Life programme are listed here: https://doi.org/10.5281/zenodo.4783585.

Members of Wellcome Sanger Institute Scientific Operations: DNA Pipelines collective are listed here: https://doi.org/10.5281/zenodo.4790455.

Members of the Tree of Life Core Informatics collective are listed here: https://doi.org/10.5281/zenodo.5013541.

Members of the Darwin Tree of Life Consortium are listed here: https://doi.org/10.5281/zenodo.4783558.
