# Supplementary material for: The genome sequence of the dark-edged bee fly, Bombylius major (Linnaeus, 1758)
Source: Wellcome Open Res. 2023 Sep 1;8:379. [Version 1] doi: 10.12688/wellcomeopenres.19804.1 (PMC10964003; doi:10.12688/wellcomeopenres.19804.1)
Supplement: Peer review history

# Reviewer report: Reviewer response for version 1

**DOI:** 10.21956/wellcomeopenres.21936.r75573

**Published:** 2024-03-25

**Authors:** Victoria E Mullin

**Affiliations:** 1 Trinity College Dublin, Dublin, Ireland

The authors describe the high-quality assembly of the dark-edge bee fly, *Bombylius major*. This genome note is well written and reports sample collection through to genome annotation. While well written, the report does not fully detail the practical applications for research of *Bombylius major*, perhaps useful for broader readership.

 The methodology used is technically sound, following the previously reported assembly and QC pipelines of the Darwin Tree of Life consortium. The assembly is a total length of 304.3 Mb, assigned to 7 chromosomal-level scaffolds and with a k-mer completeness of 99.99% and a BUSCO completeness of 94.8%.

 Data has been uploaded to ENA and includes the genome assembly of Wolbachia endosymbiont (group A) of *Bombylius major*; Specimen metadadata, sequencing information and assembly statistics are also all freely available (tolqc). It may be advantageous for future research for the mitogenome to be available on GenBank.

 This data is an important genomic resource that will enable future studies focusing on a variety of research questions including evolutionary and population genomics.

Are sufficient details of methods and materials provided to allow replication by others?

Yes

Is the rationale for creating the dataset(s) clearly described?

Yes

Are the datasets clearly presented in a useable and accessible format?

Yes

Are the protocols appropriate and is the work technically sound?

Yes

Reviewer Expertise:

ancient & historical genomics, population genomics

I confirm that I have read this submission and believe that I have an appropriate level of expertise to confirm that it is of an acceptable scientific standard.

# Reviewer report: Reviewer response for version 1

**DOI:** 10.21956/wellcomeopenres.21936.r75572

**Published:** 2024-03-25

**Authors:** Leonardo Tresoldi Gonçalves

**Affiliations:** 1 Universidade Federal do Rio Grande do Sul, Porto Alegre, Brazil

This paper presents a high-quality reference genome for the dark-edged bee fly, *Bombylius major*. The paper is well-written and the methodology for obtaining the dataset is clearly explained. I only have the following suggestions:

1. Please revise the Background section. Several of the sentences about the biology of *B. major* lack references, especially in the second paragraph.
2. The authors mentioned they used MitoHiFi to assemble the mitochondrial genome, a program that "runs MitoFinder or MITOS and uses these annotations (..)". However, it remains unclear which mode of MitoHiFi was employed for annotation – MitoFinder or MITOS. Please specify.

Are sufficient details of methods and materials provided to allow replication by others?

Yes

Is the rationale for creating the dataset(s) clearly described?

Yes

Are the datasets clearly presented in a useable and accessible format?

Yes

Are the protocols appropriate and is the work technically sound?

Yes

Reviewer Expertise:

Phylogenetics, phylogenomics, genomics, DNA barcoding.

I confirm that I have read this submission and believe that I have an appropriate level of expertise to confirm that it is of an acceptable scientific standard.
